# Update of the human early life exposome (HELIX) cohort: a European population-based exposome cohort—cohort profile

**DOI:** 10.1136/bmjopen-2026-122608

**Published:** 2026-08-20

**Authors:** Parisa Montazeri, Léa Maitre, Sandra Andrušaitytė, Kristine Bjerve Gützkow, Kateřina Coufalíková, Roberta Davidson, Lorenzo Fabbri, Regina Grazuleviciene, Mònica Guxens, Barbara Heude, Norun Hjertager Krog, Lesley Hoyles, Marianna Karachaliou, Amrit Kaur Sakhi, Hector C Keun, Jana Klánová, Christos Lionis, Katerina Margetaki, Anne L McCartney, Rosemary RC McEachan, Cristina Balcells, Tim S Nawrot, Claire Philippat, Elliott Price, Joane Quentin, Pierre Jean Saulnier, Rémy Slama, Cathrine Thomsen, Jose Urquiza, Panagiotis A Vorkas, Dagmar Waiblinger, John Wright, Tiffany C Yang, Martine Vrijheid

**Affiliations:** 1 ISGlobal, Barcelona, Spain; 2 Universitat Pompeu Fabra (UPF), Barcelona, Spain; 3 ISCIII, CIBERESP, Madrid, Spain; 4 Department of Environmental Sciences, Vytautas Magnus University, Kaunas, Lithuania; 5 Division of Environmental Health, Norwegian Institute of Public Health, Oslo, Norway; 6 RECETOX, Faculty of Science, Masaryk University, Brno, Czech Republic; 7 Catalan Institution for Research and Advanced Studies (ICREA), Barcelona, Spain; 8 Université Paris Cité and Université Sorbonne Paris Nord, Inserm, INRAE, Centre for Research in Epidemiology and StatisticS (CRESS), F-75004 Paris, France; 9 Department of Biosciences, School of Science & Technology, Clifton Campus, Nottingham Trent University, Nottingham NG11 8NS, UK; 10 University Medical Center, Erasmus MC, Rotterdam, the Netherlands; 11 Clinic of Social Preventive Medicine, Department of Social Medicine, Faculty of Medicine, University of Crete, Heraklion of Crete, Greece; 12 Division of Systems Medicine, Department of Metabolism, Digestion and Reproduction, Imperial College London, London, UK; 13 Cancer Metabolism & Systems Toxicology Group, Division of Cancer, Department of Surgery & Cancer, Imperial College London, London, UK; 14 Social Medicine, School of Medicine, University of Crete, Heraklion, Greece; 15 Born in Bradford, Bradford Institute for Health Research, Bradford Teaching Hospitals NHS Foundation Trust, Bradford, UK; 16 Centre for Environmental Sciences, Hasselt University, Hasselt, Belgium; 17 Environmental Epidemiology Applied to Development and Respiratory Health Team, Institute for Advanced Biosciences, University Grenoble Alpes, Inserm, CNRS, Grenoble, France; 18 CHU Grenoble-Alpes, Grenoble, France; 19 Clinical Investigation Centre CIC 1402, Université de Poitiers, INSERM, CHU Poitiers, Poitiers, France; 20 SMILE, Institut de Biologie de l’ENS (IBENS), Ecole Normale Supérieure, Université PSL, CNRS, INSERM, F-75005 Paris, France; 21 PARSEC, Ecole Normale Supérieure, Université PSL, CNRS, INSERM, F-75005 Paris, France

**Keywords:** EPIDEMIOLOGY, Observational Study, Adolescent

## Abstract

**Abstract:**

**Purpose:**

The Human Early Life Exposome (HELIX) cohort was established to assess a wide range of environmental exposures (the exposome) during early life and study the effects of the early life exposome on child and adolescent health outcomes and molecular omics signatures. Here, we describe the second follow-up of the HELIX cohort during adolescence, including measurements available and population characteristics.

**Participants:**

HELIX is a collaborative study across six established and ongoing population-based birth cohort studies in six European countries (France, Greece, Lithuania, Norway, Spain and the United Kingdom). The cohort includes 1663 mother-child pairs recruited during pregnancy from 2003 to 2009 and followed up during childhood at 6–12 years from 2013 to 2016. This update summarises the most recent follow-up during adolescence (12–18 years) which took place from 2020 to 2022 and included 864 participants.

**Findings to date:**

HELIX data have been used extensively in more than 100 publications, specifically pioneering the implementation of exposome approaches to address questions around the occurrence and effects of multiple exposures to real-life pollutant mixtures. These findings have improved our understanding of how multiple exposures co-exist and which exposures are driving the early-life exposome. Further, HELIX data have been used in exposome-wide discovery analyses to examine child health outcomes and used high-throughput omics techniques to characterise the internal exposome.

**Future plans:**

Although no additional follow-up visits are currently planned, HELIX will remain an active exposome research resource for years to come. The cohort will leverage its comprehensive database and extensive biobank to investigate complex exposure-omics-health relationships and measure additional biological markers.

**STRENGTHS AND LIMITATIONS OF THIS STUDY**

- *Comprehensive exposome characterisation*: The assessment of the exposome, including physical, lifestyle, social, environmental and chemical exposures during three critical windows of development, provides a lifecourse view to exposome research.
- *Integration of omics data*: Use of biological samples (blood, urine, stool) to conduct multiomics profiling provides insights into the underlying biological pathways of disease.
- *Standardised study protocols and procedures*: The development and public sharing of standardised operating procedures for data collection ensures high methodological rigour and facilitates reproducibility.
- *Loss to follow-up*: The substantial loss to follow-up in adolescence (52% cohort retention) may introduce selection bias, limit generalisability and reduce statistical power to detect true associations.
- *Generalisability:* The predominately white, European and urban cohort composition limits the generalisability of findings to other population groups.

## Introduction

 The exposome represents the totality of environmental exposures a person experiences from conception throughout life. It was introduced as a compliment to the human genome and to bring attention to the need for a more complete environmental exposure assessment.^1^ Since its introduction, the exposome has grown and expanded beyond a primary focus on external environmental pollutants to include complex interactions between social determinants, lifestyle factors and internal biological responses like gene expression, inflammation and metabolism.

The original aim of the HELIX project at its outset was to measure and describe multiple environmental exposures from the different exposome domains during early life and associate these with omics markers and child health outcomes; the study rationale and research questions have previously been described.^2 3^ Over a decade later, HELIX’s focus remains on studying the early-life exposome through the examination of exposure-omics-health associations, and the capacity to do so has now been expanded with a new follow-up during adolescence. The aim of the new follow-up was to add an additional data collection point in adolescence, enabling the cohort to generate new evidence on how a wide range of environmental exposures—individually and jointly—impact adolescent health, on how biological responses may underlie these impacts and on critical exposure periods (pregnancy, childhood, adolescence). Further, the follow-up aimed to include new data on factors that are of particular relevance in adolescents. Here, we aim to provide a detailed description of the adolescent follow-up of the HELIX cohort, including available measurements and characteristics of the study population that may be used as a resource for HELIX researchers using the data or interested in doing so in the future.

## Cohort description

The Human Early Life Exposome (HELIX) cohort represents a collaborative study across six established longitudinal population-based birth cohorts in six European countries: the Born in Bradford (BiB) study in the United Kingdom,^4^ the Étude des Déterminants pré et postnatals du développement et de la santé de l’Enfant (EDEN) study in France,^5^ the INfancia y Medio Ambiente (INMA) cohort in Spain,^6^ the Kaunas cohort (KANC) in Lithuania,^7^ the Norwegian Mother, Father and Child Cohort Study (MoBa)^8^ and the RHEA Mother Child Cohort study in Greece.^9^ Baseline recruitment dates ranged from 2003 (EDEN) to 2009 (KANC). Briefly, all recruitment took place during pregnancy during a prenatal care visit; all cohorts included at least one visit during pregnancy, one at birth and subsequent visits after delivery. All cohorts had also collected considerable data during pregnancy and birth, from children of similar age ranges and had the capacity to continue follow-up using standardised protocols.^3^

**Figure 1:**
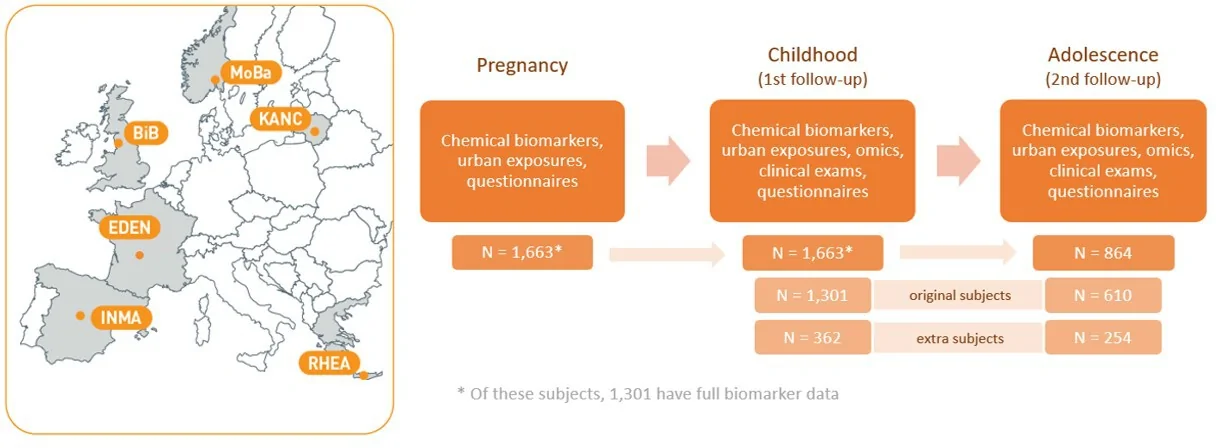
Human Early Life Exposome (HELIX) cohort study design, geographical distribution and follow-up sample sizes. Overview of the HELIX data collection time points, data domains collected and participant retention. BiB, Born in Bradford; EDEN, Étude des Déterminants pré et postnatals du développement et de la santé de L'Enfant; INMA, INfancia y Medio Ambiente; KANC, Kaunas cohort; MoBa, Norwegian Mother, Father and Child Cohort Study.

The original cohorts included over 30 000 mother-child pairs, from which a subcohort (HELIX) was selected as detailed in Maitre *et al.*^3^ In this subcohort, a follow-up visit took place between December 2013 and February 2016 when children were aged between 6 and 12 years (mean age=8 years) to assess child health outcomes and to fully characterise different areas of the exposome using common standardised protocols and questionnaires. Approximately 200 to 400 mother-child pairs participated from each cohort, totalling 1663 subjects in the subcohort. 1301 of the 1663 subjects were included in the initial exposure biomarker analysis. Biomarkers for the remaining 362 subjects that gave biological samples during the childhood visit were analysed at a later stage (figure 1). The HELIX subcohort collected data on a wide range of environmental exposures in pregnancy and childhood (urban, chemical, lifestyle, social), omics markers in childhood (methylome, transcriptome, proteome, metabolome, genome) and child health outcomes (anthropometry, cardiometabolic, neurodevelopment, respiratory), as previously documented.^3^

Visits for the new follow-up began in February 2020, but they were quickly suspended due to confinements from the COVID-19 pandemic. After a delay, visits eventually resumed with the last visits completed in December 2022. Of the 1663 children who completed the first HELIX visit (childhood), 864 subjects participated in the second follow-up (adolescence), just over half (52%) of those invited (figure 1 and table 1). Response rates were highest in INMA (74%), and lowest in MoBa (19%), with the other cohorts varying between 40% and 60% (table 1). The low response rate in some cohorts is likely due to a combination of factors, including repeated lockdown periods during the COVID pandemic, the age group (adolescents are a challenging group to engage) and General Data Protection Regulation-related restrictions that affected how contact could be made with the subjects in MoBa.

**Table 1:** Human Early Life Exposome (HELIX) cohort visit dates and sample size

Cohort	Invited (data pregnancy+childhood)	Participated (adolescent visit)	Visit dates (adolescent)
BiB, United Kingdom	231	135 (58%)	July 2021–January 2022
EDEN, France	205	80 (39%)	November 2021–January 2022
INMA, Spain	489	360 (74%)	February 2020–July 2022
KANC, Lithuania	207	115 (56%)	June 2021–May 2022
MoBa, Norway	291	56 (19%)	June 2022–August 2022
RHEA, Greece	240	118 (49%)	July 2021–July 2022
Total	1663	864 (52%)	February 2020–August 2022

BiB, Born in Bradford; EDEN, Étude des Déterminants pré et postnatals du développement et de la santé de l’Enfant; HELIX, Human Early Life Exposome; INMA, INfancia y Medio Ambiente; KANC, Kaunas cohort; MoBa, Norwegian Mother, Father and Child Cohort Study.

To ensure continuity in the cohort, many of the same exposures, omics and health outcomes measured during the childhood visit were assessed again in the adolescent follow-up described here, using the same standardised protocols (table 2). The data were collected across two visits scheduled 8 to 10 days apart; visit 1: participants were given the questionnaires (links) to complete, devices for tracking and biomonitoring kits for urine (weekly pool=6 days, 12 samples) and stool collection, and visit 2: fasting blood was collected, and study staff reviewed and collected the questionnaires, devices and biological samples. The clinical visit was done during the first or second visit, depending on the participant. During this follow-up compared with the childhood follow-up, several new questions were added to the adolescent questionnaires that specifically sought to capture the adolescent exposome, including mobile technology and screen time use, and risky lifestyle behaviours like smoking and alcohol. Given the visits aligned with the COVID-19 pandemic, an extra questionnaire was added which details COVID-19 diagnosis, testing, exposure and vaccination.

**Table 2:** Collected and available data by subcohort during the adolescent follow-up visit

	Subcohort						Total	% of total
	BIB	EDEN	KANC	MOBA	RHEA	INMA		
Maximum N	135	80	115	56	118	360	864	–
Biological samples:								
Urine	102	71	113	52	110	351	799	92.5%
Blood	107	79	110	46	96	336	774	89.6%
Stool (faeces)	59	35	88	40	74	205	501	58.0%
General questionnaire:	135	80	115	56	118	360	864	100.0%
Clinical examination	126	80	115	56	118	358	853	98.7%
Neurodevelopment	134	80	115	56	110	360	855	99.0%
Spirometry	95	56	62	42	0	197	452	52.3%
Sensors	72	44	112	37	91	297	653	75.6%
Exposures:								
Chemicals in blood	101	70	108	46	91	333	749	86.7%
Chemicals in urine	99	56	113	52	100	341	761	88.1%
Outdoor (using QGIS)	125	74	114	54	106	330	803	92.9%
Omics:								
Metabolomics in blood	107	70	109	46	95	334	761	88.1%
Metabolomics in urine	99	56	113	52	99	337	756	87.5%
Proteomics (cardiometabolic)	105	69	110	46	93	333	756	87.5%
Proteomics (Olink)	96	67	81	45	90	323	702	81.3%
Telomere length	99	68	108	45	91	333	744	86.1%
Metagenomics (gut microbiome)	59	35	88	40	73	205	500	57.9%

BiB, Born in Bradford; EDEN, Étude des Déterminants pré et postnatals du développement et de la santé de L'Enfant; INMA, INfancia y Medio Ambiente; KANC, Kaunas cohort; MoBa, Norwegian Mother, Father and Child Cohort Study; RHEA, The Rhea Study (The Mother-Child Cohort in Crete, Greece (Rhea Study)).

Several exposure assessments were improved compared with the previous visit. Adolescents were asked to wear personal sensors for 1 week, including EXPOApp—an app installed on mobile phones for collection of Global Positioning System (GPS) and physical activity data,^10^ GENEActiv—wrist accelerometers measuring sleep and physical activity data^11^ and ActiGraph (in a subset)—accelerometer worn on the waist to collect physical activity data.^12^ Combined, these sensors provide detailed personal data on mobility and microenvironments, physical activity and sleep. Further, adolescents provided fasting blood samples during the clinical visit to enable accurate measurement of biomarkers relevant to outcomes such as cardiometabolic syndrome and metabolic dysfunction-associated steatotic liver disease. These blood samples were also used to measure leucocyte telomere length, permitting the study of telomere length rank change from childhood to adolescence, and protein levels, providing repeat measures in childhood and adolescence. Detailed protocols and standard operating procedures have been made available publicly (https://athleteproject.eu/toolbox/). They cover all aspects of data collection including fieldwork protocols, biological sample collection, device use, clinical measurements, urban exposure calculations, questionnaires and general consent forms. Tables 3–5 provide a detailed list of all measurements (exposures, omics and outcomes) in the HELIX cohort derived from the visits. Data from this visit have been cleaned by HELIX researchers involved in the project. Data cleaning consisted of detection and handling of data anomalies and outliers, data imputation (only for chemicals below the limit of detection and omics data), generation of new summary variables, calculation of curves (spirometry) and z-scores (body mass index, blood pressure) and language translation to English.

**Table 3:** Exposure data collected by time period

Exposure group	Exposure variables	Data collection method/matrix	Pregnancy	Childhood	Adolescence
Chemical exposures					
Perfluoroalkyl substances (PFASs)	Up to 25 PFASs including PFOS, PFOA, PFNA, PFUNDA, PFHxS	Plasma	X	X	X
Persistent organic pollutants (POPs)	DDE, DDT, HCB, PCBs, brominated flame retardants	Serum	X	X	–
Metals and elements	Up to 16 metals and elements	Whole blood	X	X	X
Phthalate and DINCH metabolites	15 including high and low molecular weight	Urine	Spot sample	Pool of two spot samples	Weekly pool
Phenols	Four parabens, 5 phenols, BP3 and triclosan	Urine	Spot sample	Pool of two spot samples	Weekly pool
Organophosphate pesticide metabolites	Six dialkyl phosphate metabolites	Urine	Spot sample	Pool of two spot samples	Weekly pool
Organophosphate flame-retardant metabolites	DPHP	Urine	–	–	Weekly pool
Specific pesticide and pyrethroid metabolites	TCPy, 3-PBA, 2,4-D	Urine	–	–	Weekly pool
Glycol ether metabolites	PhAA, BAA	Urine	–	–	Weekly pool
Polycyclic aromatic hydrocarbon (PAH) metabolites	ten metabolites including, 1-OH-PYR and 3-OH-CRY	Urine	–	–	Weekly pool
Exogenous molecules	Untargeted screening	HR/MS	–	–	X
Ambient and urban exposures					
Outdoor air pollution	NO2, PM2.5, PM10	Air pollution models (ELAPSE/ESCAPE)	X	X	X
Personal air pollution	NO2	Diffusion tubes	–	X	X
Indoor air pollution	NO2, PM2.5, PMabs, benzene, toluene, xylene, ethylbenzene	Diffusion tubes and prediction model	–	X	–
Road traffic noise	Noise levels	Regulatory noise maps	X	X	X
Built environment	Population and building density, street connectivity, facility density, land use, walkability	Land cover/use maps	X	X	X
Natural spaces including green spaces	Surrounding greenness, distance to green/blue spaces	Remote sensing and land cover/use maps	X	X	X
Traffic and transport	Traffic load, distance to roads, public transport network	Land use maps	X	X	X
Social deprivation	Area level indicators	National deprivation indicators	X	X	X
Food environment	Fast food restaurants, healthy food places	Facilities maps	–	–	X
Meteorology	Temperature, wind, humidity, pressure	Daily average from city monitoring	X	X	X
Ultraviolet (UV)	Ambient UV radiation levels	Remote sensing	X	X	X
Light	Night-time light	Remote sensing	X	X	X
Commuting routes	Routes and modes	QGIS	–	X	X
Mobility	Time spent in different microenvironments	EXPOApp	–	X (subgroup)	X
Lifestyle and social exposures					
Smoking	Self-reported smoking, cotinine	Questionnaire/cotinine (urine)	Smoking during pregnancy, cotinine	Passive smoking, cotinine	Smoking (tobacco, e-cig, hashish, passive)
Physical activity	Duration and intensity and sedentary activities	Questionnaires, Actigraph, EXPOApp	–	X	X
Sleep	Sleep disturbances and duration	Questionnaires, GeneActiv	–	X	X
Technology	Mobile technology use/screen time	Questionnaire	–	–	X
Diet	Diet quality, FFQ	Questionnaire	X	X	X
Social and economic capital	Parental education, family affluence score, social contact, social participation, house crowding	Questionnaire	X	X	X
Psychosocial	Stress, cortisol	Questionnaire (Q), hair (H), urine (U)	X (Q)	X (Q, H, U)	X (Q)
X=measurement available, -=measurement not available, Q=questionnaire, H=hair					

BAA, butoxyacetic acid; BP3, benzophenone-3; 2,4-D, 2,4-Dichlorophenoxyacid; DDE, dichlorodiphenyldichloroethylene; DDT, dichlorodiphenyltrichloroethane; DINCH, diisononyl cyclohexane-1,2-dicarboxylate; ELAPSE, Effects of Low-Level Air Pollution: A Study in Europe; ESCAPE, European Science Cluster of Astronomy & Particle physics ESFRI research infrastructures; FFQ, food frequency questionnaire; HCB, hexachlorobenzene; HR/MS, high-resolution mass spectrometry; 3-OH-CRY, 3-Hydroxychrysene; 1-OH-PYR, 1-Hydroxyphenanthrene; PAHs, polycyclic aromatic hydrocarbons; 3-PBA, 3-Phenoxybenzoic acid; PFASs, perfluoroalkyl substances; PFHxS, perfluorohexane sulfonic acid; PFNA, perfluorononanoate; PFOA, perfluorooctanoate; PFOS, perfluorooctane sulfonate; PFUNDA, perfluoroundecanoic acid; PhAA, phenoxyacetic acid; PM, particulate matter; TCPy, 3,5,6-Trichloro-2-pyridinol; UV, ultraviolet.

**Table 4:** Omics data collected by time period

Omics group	Matrix	Childhood—platform	Adolescence—platform
Proteomics—targeted (cardiometabolic)	Plasma	Luminex kits: Cytokine 30-plex, Apoliprotein 5-plex, Adipokine 15-plex	Luminex kits: Cytokine 30-Plex Human Panel, R&D Systems custom
Proteomics	Plasma	Olink proteomics platforms (Explore Inflammation Panel I and Explore Cardiometabolic Panel I)	Olink proteomics platforms (Explore Inflammation Panel I)
Methylation	Buffy coat	450K, Illumina	–
Transcriptomics	Whole blood	HTA V.2.0, Affymetrix	–
MicroRNA	Whole blood	SurePrint Human miRNA rel 21, Agilent	–
Metabolomics	Serum	AbsoluteIDQ p180 kit, Biocrates	HR-LC-MS (UPLC-QTOF)
Metabolomics	Plasma	–	GC-Orbitrap MS
Metabolomics	Urine	1H NMR spectroscopy	HR-LC-MS (UPLC-QTOF)
Metagenomics (gut microbiome)	Stool	–	Shot-gun metagenomics
Genetics	Buffy coat	GSA, Illumina (+HRC imputation)	–
Telomere length	Buffy coat	Quantitative real-time PCR	Quantitative real-time PCR

GC, gas chromatography; GSA, global screening array; HRC, haplotype reference consortium; HR-LC-MS, high-resolution liquid chromatography mass spectrometry; MS, mass spectrometry; NMR, nuclear magnetic resonance; PCR, polymerase chain reaction; RNA, ribonucleic acid; UPLC-QTOF, Ultra-performance liquid chromatography-quadrupole time-of-flight mass spectrometry.

**Table 5:** Health outcome data collected by time period

Variable domain	Data collection method	Health outcomes	Childhood	Adolescence
Neurodevelopment	Cognitive tests	Cognitive function; Raven, N-back, Cups Task	X	X
	Questionnaire	Emotional and behavioural symptoms; KIDSCREEN-27, Child Behaviour Checklist (CBCL), Perceived Stress Scale 4 (PSS-4)	X	X
Cardiometabolic	Clinical exam	Growth and obesity; height, weight, BMI, waist circumference, waist-to-height ratio, bioimpedance fat mass	X	X
	Serum samples	Cardiometabolic markers; lipids, glucose, insulin, liver biomarkers	X	X
	Clinical exam	Systolic and diastolic blood pressure, heart rate	X	X
Respiratory	Questionnaire	Asthma, wheezing, rhinitis, eczema, food allergies, common infections	X	X
	Spirometry	Lung function; forced expiratory volume in 1 s (FEV1), forced vital capacity (FVC)	X	X

BMI, body mass index.

Further, the adolescent visit adds several new areas of research:

### Characterisation of the adolescent chemical exposome through targeted and non-targeted biomonitoring assays

The adolescent visit relied on a combination of targeted and non-targeted biomonitoring approaches to measure a wide range of exogenous and endogenous compounds in blood and urine samples of adolescents. These measures not only enable the study of health effects of well-established chemical pollutants but also of yet to be established or thus far unknown components of the chemical exposome and of the metabolome.

#### Targeted assays

New, highly sensitive multiassay techniques were carried out to determine concentrations of many chemical pollutants simultaneously in small volumes of biosample. These methods were developed to capture a broader range of chemicals compared with the previous time points and to include new chemicals that are increasingly used to replace chemicals that are now banned or under stricter regulation (eg, bisphenol A replaced by bisphenol S and F, phthalates replaced by di(isononyl) cyclohexane-1,2-dicarboxylate, long fluorocarbon chain polyfluoroalkyl substances (PFAS) replaced by shorter-chain PFAS. New chemical groups were also included in the adolescent follow-up, including metabolites of organophosphorus flame retardants, polycyclic aromatic hydrocarbons, pyrethroids and glycol ethers. In total, nine different compound groups and 85 different targeted compounds were analysed in the new follow-up using ultra high-performance liquid chromatography coupled to mass spectrometry (for most of the compound groups) and Inductively Coupled Plasma Mass Spectrometry (for metals/elements). Participants in the adolescent visit provided repeat urine samples over 1 week (two samples per day), instead of one spot sample, allowing for a novel within-subject pooling approach that achieves greater accuracy in the estimation of longer-term exposure to non-persistent chemical pollutants, such as phenols and phthalates.^13^

#### Untargeted assays

Recent advances in untargeted metabolomics using high-resolution mass spectrometry have paved the way for the development of methods able to provide in-depth characterisation of both the internal chemical exposome and the endogenous metabolome, simultaneously^14^ . In the adolescent follow-up, HELIX generated cutting-edge deep metabolome data for blood, serum and plasma samples and urine weekly pooled samples, using untargeted profiling by ultra-high-performance liquid chromatography coupled to high-resolution mass spectrometry (UHPLC-HR-MS; serum and urine) and gas chromatography coupled to high-resolution mass spectrometry (plasma). For UHPLC-HR-MS, a novel Sequential Window Acquisition of all Theoretical Mass Spectra approach enabling unbiased tandem mass spectrometry (MS/MS) on every sample was used. This generated metabolome data at unprecedented depth, yielding thousands of features characterised at MS/MS level, as compared with previous metabolomics studies in adolescents. This, alongside the incorporation in analyses of a comprehensive authentic standards library, supported annotation of the endogenous metabolome with elevated confidence.

Untargeted HR-MS techniques are important for their capability to screen for exogenous and endogenous molecules without a priori knowledge of their involvement in the studied outcome. Their capability for generating a vast amount of data renders them an invaluable resource for the project, which can be mined and interrogated against different outcomes and research questions, annotating novel features ad hoc for years to come.

Finally, to further enable future reusability of the generated datasets and their potential to be intercomparable or harmonised with other existing or emerging metabolomics datasets, we used a comprehensive strategy, allowing external quality assessment. This was achieved by employing widely used National Institute of Standards and Technology Standard Reference Materials. These constitute commercially available serum and urine pooled samples of certified composition of endogenous and exogenous molecules.

### Evaluating associations between the exposome, the gut microbiome and health, through shotgun metagenomics

During the adolescent visit, study participants provided stool samples collected at home, from which DNA extracts were shotgun metagenome sequenced to at least 6 billion base pairs (giga-base pairs) (6×10^9^ base pairs (bp), 150 bp paired-end) raw sequence data per sample. After removal of human DNA sequences, this large-scale resource of metagenomic (gut microbiome) sequence data was assigned to taxonomies (bacteria, archaea, viruses, fungi), providing relative abundance measures of taxa present. Genes encoded within metagenomic data were predicted and annotated, allowing functional characterisation to pathway (Kyoto Encyclopedia of Genes and Genomes) and ontology (Clusters of Orthologous Groups) levels. Lastly, metagenome-assembled genomes (MAGs) were generated from the total dataset, resulting in an adolescent gut MAG catalogue with over 7000 high-quality MAGs.

The combined analysis of metagenomics and metabolomics in the same subjects will allow for the comprehensive study of the adolescent gut microbiome and enable more precise understanding of routes of exposure (eg, diet) and of longitudinal associations between the exposome and markers of metabolic disease, immune-metabolism, microbiome function and neurological biochemistry.

## Patient and public involvement

There was no patient or participant involvement in this study design or follow-up. The six cohorts that participate in HELIX recruited healthy pregnant women and their children, who were later followed up when they were aged 6–11 years and again at 12–18 years. Each cohort made local efforts to keep their families involved in the years between HELIX visits through cohort-specific study visits, newsletters, family meetings and open days. HELIX results have been disseminated to relevant stakeholders.

## Adolescent follow-up characteristics

The distribution of sociodemographic characteristics of the participants who completed the adolescent follow-up visit was relatively similar to those of participants in the childhood follow-up visit (table 6), with cohort being the main characteristic that differed due to the differences in participation rates. The HELIX cohort remains a cohort of participants where the majority have parents who are both from the cohort country (with the exception of BiB), with high family affluence and maternal education level and with neither parent smoking during childhood (table 6).

**Table 6:** Sociodemographic characteristics for Human Early Life Exposome (HELIX) cohort participants

Characteristic	Baseline- pregnancy/childhood (n=1663)	Participants followed-up at adolescence (n=864)	Participants lost to follow-up at adolescence (n=799)	P value *
	N (%)	N (%)	N (%)	
Cohort (geography)				*<0.0001*
BiB, United Kingdom	231 (13.9)	135 (15.6)	96 (12.0)	
EDEN, France	205 (12.3)	80 (9.3)	125 (15.6)	
INMA, Spain	489 (29.4)	360 (41.7)	129 (16.1)	
KANC, Lithuania	207 (12.4)	115 (13.3)	92 (11.5)	
MoBa, Norway	291 (17.5)	56 (6.5)	235 (29.4)	
RHEA, Greece	240 (14.4)	118 (13.7)	122 (15.3)	
Parent’s country of origin (pregnancy)				*<0.0001*
Both parents native	1338 (80.5%)	673 (77.9%)	665 (83.2%)	
One parent native	107 (6.4%)	77 (8.9%)	30 (3.8%)	
Neither parent native	173 (10.4%)	91 (10.5%)	82 (10.3%)	
*Missing*	*45* (*2.7%*)	*23* (*2.7%*)	*22* (*2.8%*)	
Maternal age at childbirth	(n=1591) 30.9 (4.8)	(n=815) 31.0 (4.6)	(n=776) 30.8 (5.0)	*0.292*
Maternal education (pregnancy)				*0.673*
Low	237 (14.3%)	123 (14.2%)	114 (14.3%)	
Middle	549 (33.0%)	273 (31.6%)	276 (34.5%)	
High	773 (46.5%)	403 (46.6%)	370 (46.3%)	
*Missing*	*104* (*6.3%*)	*65* (*7.5%*)	*39* (*4.9%*)	
Maternal smoking (pregnancy)				*0.979*
No	1285 (77.3%)	660 (76.4%)	625 (78.2%)	
Yes	276 (16.6%)	142 (16.4%)	134 (16.8%)	
*Missing*	*102* (*6.1%*)	*62* (*7.2%*)	*40* (*5.0%*)	
Child sex (birth)				*0.115*
Female	768 (46.2%)	415 (48.0%)	353 (44.2%)	
Male	895 (53.8%)	449 (52.0%)	446 (55.8%)	
Socioeconomic position-FAS (childhood)				*0.244*
Low	172 (10.3%)	78 (9.0%)	94 (11.8%)	
Middle	607 (36.5%)	319 (36.9%)	288 (36.0%)	
High	840 (50.5%)	424 (49.1%)	416 (52.1%)	
*Missing*	*44* (*2.6%*)	*43* (*5.0%*)	*1* (*0.1%*)	
Parental smoking (childhood)				*0.433*
Neither parent smokes	974 (58.6%)	504 (58.3%)	470 (58.8%)	
One parent smokes	452 (27.2%)	224 (25.9%)	228 (28.5%)	
Both parents smoke	179 (10.8%)	84 (9.7%)	95 (11.9%)	
*Missing*	*58* (*3.5%*)	*52* (*6.0%*)	*6* (*0.8%*)	

* P value was calculated using the χ2 test for all categorical variables and T-test for continuous variables. The p value shown excludes the *missing* category.

BiB, Born in Bradford; EDEN, Étude des Déterminants pré et postnatals du développement et de la santé de l’Enfant; FAS, family affluence scale; HELIX, Human Early Life Exposome; INMA, INfancia y Medio Ambiente; KANC, Kaunas cohort; MoBa, Norwegian Mother, Father and Child Cohort Study.

Comparison of characteristics of the participants between the childhood and adolescent time points shows that adolescents were more likely to have a poor Mediterranean Diet Score, spent more time in sedentary activities and were more likely to have overweight/obesity (20.6% in childhood, 30.8% in adolescence) (table 7). Other characteristics showed little difference. 17.6% of adolescents reported having smoked tobacco and 22.7% having used E-cigarettes (table 7).

**Table 7:** Cohort characteristics at childhood and adolescent follow-up visits

Characteristic	Childhood (max n=1663)	Adolescence (max n=864)
Age	8.17±1.56	15.01±1.34
Has smoked tobacco	*Not measured*	
No	–	642 (74.3%)
Yes	–	152 (17.6%)
*Missing*	–	*70* (*8.1%*)
Has smoked E-cig	*Not measured*	
No	–	604 (69.9%)
Yes	–	196 (22.7%)
*Missing*	–	*64* (*7.4%*)
Mediterranean Diet Score		
Good	297 (17.9%)	157 (18.2%)
Average	1177 (70.8%)	439 (50.8%)
Poor	137 (8.2%)	213 (24.7%)
*Missing*	*52* (*3.1%*)	*55* (*6.4%*)
Asthma, doctor diagnosed		
No	1490 (89.6%)	730 (84.5%)
Yes	170 (10.2%)	96 (11.1%)
*Missing*	*3* (*0.2%*)	*38* (*4.4%*)
Food allergy		
No	1499 (90.1%)	756 (87.5%)
Yes	161 (9.7%)	86 (10.0%)
*Missing*	*3* (*0.2%*)	*22* (*2.5%*)
BMI, WHO (categorical)		
Normal weight	1321 (79.4%)	587 (67.9%)
Overweight/obesity	342 (20.6%)	266 (30.8%)
*Missing*	*0*	*11* (*1.3%*)
Systolic blood pressure, mm Hg	99.9±11.1	109.4±12.0
*Missing*	*9* (*0.5%*)	*10* (*1.2%*)
Diastolic blood pressure, mm Hg	58.7±9.8	66.0±9.0
*Missing*	*9* (*0.5%*)	*10* (*1.2%*)
Behavioural and emotional problems (total CBCL score)	24.7±19.3	23.0±19.6
*Missing*	*12* (*0.7%*)	*22* (*2.5%*)
Physical activity (MVPA) (minutes/day)	49.3±40.3	52.8±49.5
Sedentary activity (minutes/day)	227.6±123–0	460.5±137.1

Italic data referes to frequencies for missing participant data.

BMI, body mass index; CBCL, Child Behaviour Checklist; E-cig, electronic cigarette; max, maximum; MVPA, moderate to vigorous physical activity; N, sample size; WHO, World Health Organization.

## Findings to date

HELIX data from the previous childhood follow-up have been used extensively in more than 100 publications, specifically pioneering the implementation of exposome approaches to address questions around the occurrence and effects of multiple exposures to real-life pollutant mixtures. These findings have improved our understanding of how multiple exposures correlate and co-exist,^15 16^ how multiple exposures vary geographically and temporally^13 17 18^ and which social, urban and dietary factors drive parts of the early-life exposome.^18–21^ Also, HELIX data have been used in exposome-wide discovery analyses assessing simultaneously the associations between many environmental risk factors and pregnancy and child health outcomes (eg, birth weight, blood pressure, lung function, obesity, neurobehavioural, cognition, allergies^22–28^ and in studies assessing the joint effects of urban exposure clusters^29^ or mixtures of chemical pollutants^30–32^). Likewise, HELIX used high-throughput omics techniques to characterise the internal part of the exposome and to identify biological signatures and pathways that respond to and interact with environmental exposures.^33–40^ Such information may be used to develop novel exposure biomarkers, improve biological plausibility of associations, understand how different exposures may act on common or diverse pathways and, ultimately, predict environmental health-related disease before its clinical manifestation.^41^

Data from the adolescent follow-up will add important new insights into which environmental exposures are relevant to health trajectories. Trajectories from infancy to adolescence strongly determine later disease and ageing trajectories and contribute to the study of the natural course of various chronic diseases. As such, their study may suggest measures to prevent or modify the course of the disease. Further, adolescence is a time of rapid growth, adaptation and maturation of many organ systems, including the reproductive and endocrine system, but also the metabolic, respiratory, immune and nervous systems. Having two follow-up time points after pregnancy and birth uniquely enables HELIX to create these longitudinal health trajectories and further explore the effects of multiple exposures on multiple outcomes. The new data have already been used to study dietary patterns in relation to child and adolescent obesity^42^ and multiomics profiles that characterise child and adolescent obesity and metabolic dysfunction.^43^

## Strengths and limitations

The HELIX birth cohort is novel in that it has collected extensive data on over 100 environmental exposures at three different time points, as well as child and adolescent health outcomes and molecular omics data, across six European locations. Strengths of the cohort and its latest follow-up point include first the repeated measurement of exposures and health outcomes, enabling the longitudinal study of the early-life exposome and its impact on health up to adolescence. Second, by focusing on a broad range of environmental exposures that are widespread in the general population, together with information on living and social environments and on personal habits and behaviours, the cohort offers unique data to study the complexities of the exposome. Thirdly, the availability of omics measurements across several molecular layers allows the study of multiomics and cross-omics responses to environmental exposures. Lastly, the exposome was measured through a complementary set of targeted and untargeted biomonitoring measures, personal monitoring, geospatial modelling and questionnaires, aimed at obtaining solid exposure estimates for several exposome domains and at the exploration of the “unknown” part of the chemical exposome.

Weaknesses include loss to follow-up, a common issue in cohort studies. Participation varied across cohorts due to several factors, but was lowest in MoBa given the strict contact policies in Norway. As this may contribute to selection bias, and to compensate for this HELIX has developed an inverse probability weighting approach that researchers can apply to their papers (https://github.com/isglobal-cep/athlete-followup/blob/main/docs/report.pdf). Further, the HELIX cohort mainly includes data from primarily white European participants living in urban areas, limiting the generalisability of results to other population groups. However, the location of the contributing cohorts, as well as the selection of urban participants, allowed for the characterisation of the urban exposome.

## Collaboration

Researchers interested in using HELIX data can apply by completing a proposal for a specific manuscript they wish to pursue. Proposals are reviewed and decisioned by the HELIX Project Evaluation Committee. Applying researchers must be with an institute with competence in conducting research projects and that has agreed to be responsible for the conduct of the proposed manuscript. Junior researchers must have a scientific supervisor, and all proposed manuscripts must have a principal investigator with scientific responsibility for the project. For each accepted proposal, a data transfer agreement must be signed between the cohorts participating in HELIX (represented by ISGlobal) and the receiving institution. Restrictions may apply to researchers and institutions located outside of Europe, due to GDPR restrictions. HELIX encourage the participation of all interested researchers at all levels of their career.

## Data Availability

Data are available upon reasonable request.
