# Supplementary material for: Update of the human early life exposome (HELIX) cohort: a European population-based exposome cohort—cohort profile
Source: BMJ Open. 2026 Aug 20;16(8):e122608. doi: 10.1136/bmjopen-2026-122608 (PMC13504928; doi:10.1136/bmjopen-2026-122608)
Supplement: Supplementary file 1 [file bmjopen-2026-122608.reviewer_comments.pdf]

PEER REVIEW HISTORY

BMJ Open publishes all reviews undertaken for accepted manuscripts. Reviewers are asked to complete a checklist review form and are provided with free text boxes to elaborate on their assessment. These free text comments are reproduced below.

ARTICLE DETAILS

Title (Provisional)

Cohort profile: Update of the Human Early Life Exposome (HELIX) cohort: A European population-based exposome cohort

Authors

Montazeri, Parisa; Maitre, Léa; Andrušaitytė, Sandra; Bjerve Gützkow, Kristine; Coufalíková, Kateřina; Davidson, Roberta; Fabbri, Lorenzo; Grazuleviciene, Regina; Guxens , Mònica; Heude, Barbara; Hjertager Krog, Norun; Hoyles, Lesley; Karachaliou, Marianna; Sakhi, Amrit Kaur; Keun, Hector C.; Klánová, Jana; Lionis, Christos; Margetaki, Katerina; McCartney, Anne L; McEachan, Rosemary RC; Balcells, Cristina; Nawrot, Tim S; Philippat , Claire; Price, Elliott; Quentin, Joane; Saulnier, Pierre Jean; Slama, Rémy; Thomsen, Cathrine; Urquiza, Jose; Vorkas, Panagiotis A; Waiblinger, Dagmar; Wright, John; Yang, Tiffany C; Vrijheid, Martine

---

VERSION 1 - REVIEW

**Reviewer**	**1**
**Name**	**Rognmo, Kamilla**
**Affiliation**	**The University of Tromsø**
**Date**	**01-Jun-2026**
**COI**	**None**

Thank you for the opportunity to review this cohort profile of the HELIX cohort. It is a very well written and comprehensive cohort profile of a unique, cross-national cohort consisting of extensive and high-quality data. I have a few specific comments that may clarify some aspects, please see below.

Overall: The cohort is extensively described, however a table outlining the variables available in the project, data collection methods and timing of the data collections of the various cohorts should be included in the manuscript. The purpose of the cohort profile is also not clear from the text – is it meant to be of help to researchers using data or interested in using data from the HELIX cohort? If that is the case, more detailed information about the study is needed, preferably in a table, outlining the details of the study necessary for researchers using the data.

Specific comments to the manuscript:

Page 6, line 11. Faces should probably be feces.

Page 8, line 36. It should be noted in the text that the follow-up referred to in the sentence is the study being described in the submitted manuscript.

Page 11, line 20. Is the headline on the same level as the headlines denoted 1(and 2) above? If so, should this be denoted 3)? All in all, it is unclear what is being described under the heading starting with "Characterization of the adolescent chemical exposome through targeted and nontargeted biomonitoring assays" – what is the purpose of the text under this heading?

Page 16, lines 6-13. Ethical approval may be more detailed explained, in order for the cohort profile description to be easily used by researchers using the data in studies.

**Reviewer**	**2**
**Name**	**Nyakato, Patience**
**Affiliation**	**University of Cape Town, School of public health**
**Date**	**08-Jun-2026**
**COI**	**None**

---

1. How does this cohort compare to tother existing birth cohorts?

2. Are there any significant differences between participants who were retained and those who were lost to follow up? I see the table showing their demographic characteristics but were there statistical differences between them that would point towards potential selection bias?

3. Any additional information on how missing data was handled, quality control of the laboratory samples, completeness of biological samples across countries etc would strengthen the manuscript.

VERSION 1 - AUTHOR RESPONSE

Reviewer: 1
Dr. Kamilla Rognmo, The University of Tromsø
Comments to the Author:

Thank you for the opportunity to review this cohort profile of the HELIX cohort. It is a very well written and comprehensive cohort profile of a unique, cross-national cohort consisting of extensive and high-quality data. I have a few specific comments that may clarify some aspects, please see below.

Overall: The cohort is extensively described, however a table outlining the variables

available in the project, data collection methods and timing of the data collections of the various cohorts should be included in the manuscript. The purpose of the cohort profile is also not clear from the text – is it meant to be of help to researchers using data or interested in using data from the HELIX cohort? If that is the case, more detailed information about the study is needed, preferably in a table, outlining the details of the study necessary for researchers using the data.

Thank you for taking time to provide your review. The aim of the paper is to serve as a reference for those working with HELIX data and those who are looking for more information, as such we have expanded the last sentence of the "Introduction" to make the purpose of the paper more clear.

"*Here, we aim to provide a detailed description of the adolescent follow-up of the HELIX cohort, including available measurements and characteristics of the study population that may be used as a resource for researchers using the data or interested in doing so in the future.*"

In regards to more detailed information using a table that includes variables, methods, and timing: With this paper we aim to only describe the most-recent adolescent visit as the pregnancy and childhood visits were previously described in another BMJ paper published in 2018. HELIX has thousands of variables given the depth of exposures HELIX covers, so it would not be practical to include them all here is this paper, rather we have chosen to list them by group with main variable topics. These can be found in Tables Table 3, 4 and 5. In each table we have included the time-point in which it was measured, the method, and other details as necessary (e.g. matrix for omics measures).

Based on your comments and those of Reviewer 2, we have also added a new table (Table 2) detailing the sample size of data collected by variable group and reported the numbers broken down by subcohort. We have also added details to Table 5 (health outcomes measured), and additional information regarding the visit to the last 2 paragraphs of the section "*Cohort description*".

Specific comments to the manuscript:
Page 6, line 11. Faces should probably be feces.

Yes, thank you for catching this. We have changed this to "stool." It better aligns with the wording we used elsewhere is the article.

Page 8, line 36. It should be noted in the text that the follow-up referred to in the sentence is the study being described in the submitted manuscript.

We have modified this sentence, see below.

"To ensure continuity in the cohort, many of the same exposures and health outcomes measured during the childhood visit were assessed again in the follow-up described in this paper (during adolescence),..."

Page 11, line 20. Is the headline on the same level as the headlines denoted 1(and 2) above? If so, should this be denoted 3)? All in all, it is unclear what is being described under the heading starting with "Characterization of the adolescent chemical exposome through targeted and nontargeted biomonitoring assays" – what is the purpose of the text under this heading?

The section indicated "*Evaluating associations between the exposome, the gut microbiome, and health, through shotgun metagenomics*" is separate from the section above, "*Characterization of the adolescent chemical exposome through targeted and non-targeted biomonitoring assays*". The sections denoted with numbers; *1) Targeted assays* and *2) Untargeted assays*. Are sub-sections under "*Characterization of the adolescent chemical exposome through targeted and non-targeted biomonitoring assays*". We denoted them with numbers to indicate that they were subsections to that section.

Page 16, lines 6-13. Ethical approval may be more detailed explained, in order for the cohort profile description to be easily used by researchers using the data in studies.

Under the "Ethics approval" section we have added the approving ethical committee (centre) and reference approval number for each subcohort in HELIX.

Reviewer: 2
Dr. Patience Nyakato, University of Cape Town
Comments to the Author:

1. How does this cohort compare to to other existing birth cohorts?

The greatest difference between HELIX and other birth cohorts is HELIX´s focus on the exposome. Unlike traditional birth cohorts that isolate single variables or one data domain, HELIX measures the "exposome," which captures hundreds of environmental exposures from pregnancy onward. HELIX gathered data on the external exposome (e.g. built environment, climate, air pollution...) and the internal exposome (e.g. lifestyle, diet, chemical...), and incorporated omics profiling to study, exposure – omics – health associations. HELIX did this across 6 European countries and 3 critical windows of development. This quantity of high-quality data across varying time points, countries, and exposure groups makes HELIX unique.

In the last paragraph of *"Findings to date"* we included a sentence (see below), which highlights HELIX´s novelty in including multiple time points.

> *"Having two follow-up time points after pregnancy and birth uniquely enables HELIX to create these longitudinal health trajectories, and further explore the effects of multiple exposures on multiple outcomes."*

Additionally, we have changed the language of the first sentence of the *"Strengths and limitations"* section to highlight the novelty of the HELIX cohort. In the same paragraph we later describe these strengths that make HELIX unique.

2. Are there any significant differences between participants who were retained and those who were lost to follow up? I see the table showing their demographic characteristics but were there statistical differences between them that would point towards potential selection bias?

I have calculated the p-values for this table (Table 6) and added them as an additional column to the table. As you can see, only significant statistical differences exist for 2 categories, cohort and parent´s country of origin. The cohort difference is due to a significant drop in participation in MoBa, which we mention already in text was due largely to their restrictions on ability to recruit to due their interpretation of the GDPR, and we discuss this loss-to-follow up limitation in the *"Strengths and limitations"* section. In the same paragraph we go on to mention an inverse probability weighting (IPW) approach that researchers can apply to their papers and is available freely on Github with the link to access. The other difference observed is due to a slightly higher participation by those parents not from the cohort country, which I view as a positive contribution and a reflection of maintaining immigrant participation.

3. Any additional information on how missing data was handled, quality control of the laboratory samples, completeness of biological samples across countries etc would strengthen the manuscript.

Based on this comment and a similar one by the other Reviewer, we have added a new table (Table 2) detailing the sample size of data collected by domain and subcohort, and added details to Table 5 (health outcomes measured). We also added information regarding the visit to the last 2 paragraphs of the section "*Cohort description*". We have chosen not to detail the QC done by the labs here, as there are different labs for each omics group and the chemicals. Each laboratory has their own QC in addition to the QC we did as a part of the data cleaning process. Detailing those here is not the aim of the paper as each omics group QC is very extensive. As specific papers are published using these datasets, they will include the detailed QC in these papers. Similarly, our papers on associations between exposures, omics, and health outcomes each include detailed descriptions on how missing data was handled. This this may vary from paper to paper, we cannot include this in the profile paper.

Reviewer: 1
If you have selected 'Yes' above, please provide details of any competing interests.: Not applicable

Reviewer: 2

If you have selected ‘Yes’ above, please provide details of any competing interests.: Not applicable

VERSION 2 - REVIEW

**Reviewer**	**1**
**Name**	**Rognmo, Kamilla**
**Affiliation**	**The University of Tromsø**
**Date**	**22-Jul-2026**
**COI**	

Thank you for making the recommended revisions, I have no further comments. The paper is well written, and the study protocol provides a thorough description of the HELIX cohort.
